# Knowledge is power - keeping radiology relevant in the age of AI-based healthcare

**DOI:** 10.1590/0100-3984.2021.54.4e2

**Published:** 2021

**Authors:** Erique Guedes Pinto

**Affiliations:** 1 Universidade da Beira Interior, Faculdade de Ciências da Saúde, Covilhã, Portugal. Email: ericguedespinto@gmail.com.

In the special article entitled “Misconceptions in the health technology industry that are delaying the translation of artificial intelligence technology into relevant clinical applications”, published in this issue of Radiologia Brasileira, the author exposes problems facing the industry that is driving the artificial intelligence (AI) revolution in healthcare^(1)^. One such problem is the misunderstanding of what AI technology is, notably not by the consumers of this novel technology but by the industry developing it. The AI revolution is disruptive by its very nature. It simulates human reasoning and the generalization of knowledge, its inner workings being clouded by ‘hidden layers’ of interconnected neuron-like mathematical structures. It is ‘magical’ not only to patients and healthcare providers but also to the developers themselves, who willingly give up control of directing the medical knowledge they do not master.

During the first attempts at developing AI, expert systems sought to reproduce the opinion of experts (i.e., a program following a set of rules defined by a panel of doctors, covering every reasonably predicted case), such systems including computer-aided diagnostic tools that calculated the risk of malignancy on the basis of the presence of calcification or poorly defined borders in a lesion. The current iteration of AI relies on the neural network architecture, based on a randomly generated initial state, derivative functions, and a back-propagation algorithm. Back-propagation training occurs as each case in the training dataset changes the state of the network slightly. Once trained, this inner state is a mathematical snapshot of the knowledge gained.

The reliability of the training data has been recognized as being crucial, because it strongly influences the quality of the results. Radiologists support the development of AI systems by acting as the source of truth (i.e., labeling datasets and measuring distances). Ironically and much like philosophers of old, no two radiologist are always in perfect agreement. Inter-observer variability is as relevant today as ever and is one of the major motivators of AI-driven healthcare. However, inter-observer variability and other biases will inevitably permeate from research laboratories into the trained AI systems and then be applied in clinical practice, being recommended by scientific societies and mandated by stakeholders such as health insurance systems. One example is the fact that the current guidelines for the management of lung nodules recommend the use of volumetric techniques^(2)^.

Ideally, there should be a deep reshaping of internal management, leadership, and innovative processes within technology companies which would allow them to adapt and thrive under the new paradigm of AI-based healthcare. However, from a business point of view, the risks for disruptive changes are high, especially in a market that is as yet poorly defined and without a stable legal or ethical framework, favoring incremental evolution and the perpetuation of old vices of the corporate economy.

Medical leadership, which is at the heart of AI-based healthcare, is needed in order to shift the focus of development back to the clinical purpose of caring for patients. However, the differences between the problem space and language specific to business, the life sciences, and the exact sciences constitute an obstacle to effective communication and collaboration. Medical reasoning is not easily applied to the exact sciences, much like analytical reasoning about matrix calculus is wasted in deciding on a surgical indication for a cancer patient. Among these groups of professionals, physicians have the most to gain from the insights of AI, although they also have the most to lose, specifically their authority and relevance as clinical decision-makers. By its technological nature, radiology will be on the front line of this paradigm shift.

Believing that AI can be stopped by radiologists who refuse to use new tools is a futile exercise in vanity. Instead, we need to adapt and embrace neural networks like the Bayes theorem once was. Only then can we truly understand the scope of challenges facing AI-based healthcare and push the technology forward. Academia will certainly play a role in this shift, because adequate skills for reasoning about AI are blatantly lacking in undergraduate medical training and only available much later in a medical career^(3)^. Medical educators need to rethink how they prepare future generations of physicians, because the latter will carry the burden of keeping AI systems in check, identifying their limitations and pitfalls, thus safeguarding the interests of patients.
